# The Annual Report of the Eastern Asylum, in the City of Williamsburg, Virginia, for 1846

**Published:** 1847-04

**Authors:** John M. Gault

**Affiliations:** Superintendent and Physician


*The Annual Report of the Eastern Asylum, in the City of Williamsburg, Virginia, for* 1846. JOHN M. GAULT, M. D., Superintendent and Physician.

*Fourteenth Annual Report of the Trustees of the State Lunatic Hospital at Worcester, Mass. Dec.* 1846.

*Eighth Annual Report of the Directors and Superintendents of the Ohio Lunatic Asylum, for the year* 1846.

*Fourth Annual Report of the Managers of the State Lunatic Asylum, made to the Legislature, (N. Y.) Feb.* 2, 1847.

We would express our obligations to the medical officers connected with the above mentioned institutions, for the interesting and valuable reports whose titles we have given. A fund of useful information is communicated to the profession and public every year by means of these stated publications. We have reason to feel proud of the scientific attention now being given in this country to that department of medicine which concerns mental disorders, and of the public liberality toward institutions for the relief of those who suffer from the terrible calamity of insanity. Each of the four institutions referred to, appears to be in a highly flourishing condition. The State Asylum at Utica continues under the sole management of Dr. Brigham. Dr. Awl is the Medical Superintendent of the Ohio Asylum. At the Worcester Hospital a change has lately taken place, Dr. Woodward having resigned, and Dr. Chandler, formerly an assistant of Dr. Woodward, and more recently at the head of the New-Hampshire Asylum, having been elected his successor. We trust that annual reports from the various lunatic institutions of our country, will be continued. They should be extensively circulated in order to sustain and promote public interest in the subject, and, after a series of years, the amount of statistical facts which will in this way accumulate, will furnish the data for important analytical results.

